# Limitations of maternal recall for measuring exclusive breastfeeding rates in South African mothers

**DOI:** 10.1186/s13006-018-0159-8

**Published:** 2018-05-25

**Authors:** Helen Mulol, Anna Coutsoudis

**Affiliations:** Department of Paediatrics and Child Health, University of KwaZulu-Natal, Durban, South Africa

**Keywords:** Maternal recall, Deuterium dilution, Stable isotope, Exclusive breastfeeding

## Abstract

**Background:**

Maternal recall is most commonly used to determine exclusive breastfeeding rates. A gold standard stable isotope method is available which can determine intake of breast milk versus water from sources other than breast milk and thus objectively determine exclusive breastfeeding. The objectives of this study were to determine exclusive breastfeeding rates by both maternal recall and the objective stable isotope method and discuss the limitations and usefulness of the two methods.

**Methods:**

The study involved 100 mother-infant pairs in a peri-urban area in Durban, South Africa and study visits took place from July 2012 to September 2014. Maternal recall of exclusive breastfeeding was carried out using the World Health Organization’s 24 hour recall of infant feeding and this was compared to the objective measurement of exclusive breastfeeding using the stable isotope technique at three time points: six weeks, three and 5.5 months. The objective measurements were carried out using two different cut off values for exclusive breastfeeding. Kappa analysis was used to quantify the relationship between maternal recall and results from the stable isotope technique for each mother-infant pair.

**Results:**

Over reporting of exclusive breastfeeding was common at the three different time points regardless of the cut off value used to assess exclusive breastfeeding by the stable isotope technique. Kappa analysis also revealed only slight or fair agreement (K < 0.24) between reported and measured exclusive breastfeeding at all time points.

**Conclusions:**

Maternal recall of exclusive breastfeeding is limited in accuracy and should be restricted to large scale epidemiological surveys. The more objective gold standard stable isotope method for measuring intake volumes of breast milk should be used to evaluate interventions with smaller representative samples.

## Background

The World Health Organization (WHO) and international agencies recommend all mothers exclusively breastfeed (EBF) their infants for the first six months. Despite this, rates of EBF in children of low- and middle-income countries remain suboptimal (a 2013 survey estimated that 37% of infants younger than six months of age are EBF) [1]. The 2003 South African Demographic and Health Survey (SADHS) carried out by the Department of Health showed low rates of EBF at time points up to six months (11.2% < 2 months; 12.2% 2–3 months; 1.3% 4–5 months; and 0.7% 6–7 months; or 8.3% over the first six months) [2]. In view of the importance of breastfeeding to improve child survival and given the historically very low EBF rates, the South African government called a Breastfeeding Consultative Forum in 2011 and committed itself to increase efforts to protect, promote and support breastfeeding [3]. Several provinces therefore renewed their efforts at implementing breastfeeding promotion and support. The KwaZulu-Natal province (KZN) in particular was very active and in 2014 the KwaZulu-Natal Initiative for Breastfeeding Support (KIBS) was established as a three year programme, which aimed to improve exclusive breastfeeding rates in the KwaZulu-Natal province, including employing lactation and nutrition advisors in hospitals and providing training for community health workers in the households [4].

The promotion of breastfeeding in South Africa was historically hampered by the high HIV prevalence in the country. KZN in particular has very high HIV prevalence rates with an antenatal HIV prevalence of 40.1% recorded in the national 2013 survey [5]. However, in 2010 with the change in WHO infant feeding guidelines for HIV infected women [6], national guidelines in South Africa followed suit encouraging six months of exclusive breastfeeding [7]. More recently again South Africa followed HIV guidelines [8] whereby HIV infected pregnant women are all provided with life-long antiretroviral treatment and women are encouraged to breastfeed exclusively for six months and then continue breastfeeding for up to 24 months.

Increasing the rate of EBF in the first six months to 50% (for both HIV infected and uninfected mothers) is one of the United Nations Decade of Nutrition’s six major global targets [9] and therefore monitoring EBF rates is important for countries. Most surveys on exclusive breastfeeding use a 24 h maternal recall of infant feeding practices to determine the rate of exclusive breastfeeding and monitor changes over time. Another method available to determine EBF rates is the deuterium dilution dose-to-mother method (DTM method). This stable isotope technique developed by Coward et al. [10] was standardized by the International Atomic Energy Agency (IAEA) [11] and enables determination of breast milk intake volumes and intake of water from sources other than breast milk. This then allows exclusivity of breastfeeding to be determined objectively, which can then be compared to EBF data obtained from maternal recall. A few studies have been undertaken in Bangladesh [12], Botswana [13], Cameroon [14] and India [15] that have compared EBF from maternal recall and the stable isotope technique.

This study was set up to determine the EBF rates using maternal recall as well as the more objective stable isotope method. A further objective of the study was to investigate/discuss the limitations and usefulness of the two methods for monitoring EBF rates.

## Methods

### Study design

The study design was longitudinal and observational and was carried out at Cato Manor Clinic, also known as the Umkhumbane Community Health Centre, in Cato Manor, Durban, South Africa. Mothers could access the study site easily when coming from clinic visits and the clinic was located centrally in the community from which the participants were drawn. The study being reported on in this manuscript is a sub-study of a larger study whose main objective was to determine breast milk intake/output volumes at five different time points over 12 months during the period from July 2012 to September 2014 [16]. The sample size calculated to be adequate to answer this research question was 100 mother-infant pairs. The sub-study only included relevant data that was available for the first three time points (infant age of 6 weeks, 3 and 5.5 months).

In order to be eligible for inclusion in the parent study [16], and therefore the sub-study, mothers had to be participating in the Improved Nutrition Program that was taking place concurrently at the Cato Manor Clinic. This was a program that provided approximately 16 breastfeeding and nutrition training sessions to mothers over a period of 12 months [16]. Other eligibility criteria for mothers included the following: well (no clinical symptoms or medical history of cardiac or other chronic conditions); no HIV or other infectious disease; intends to breastfeed her baby for 12 months; intends to live in the neighborhood of the recruitment clinic for 12 months after delivery; and African. However, recruitment was slow, and an amendment was approved by the Biomedical Research Ethics Administration of the University of KwaZulu-Natal (UKZN-BREC) in March 2013 that allowed asymptomatic HIV infected mothers to also participate in the study. This resulted in recruitment of 40 HIV infected and 60 HIV uninfected mothers.

Exclusion criteria for the mother were: pregnancy; and BMI < 18.5 kg/m^2^.

Inclusion criteria for the infant were: full term; and birth weight > 2.3 kg.

Exclusion criteria for the infant were: twins; any defect that interferes with feeding; and chronic illness e.g. congenital heart disease, cerebral palsy.

### Determination of EBF using maternal recall

A WHO 24 h recall questionnaire [17] was used to determine infant feeding by maternal recall at each study visit. Classification of exclusive breastfeeding at the first three time points of the study, viz. infant age of 6 weeks, 3 months and 5.5 months was done according to the WHO definition [18] of breast milk, vitamins and mineral supplements and medicine only.

### Determination of EBF using the DTM method

To determine exclusivity of breastfeeding, the DTM method was carried out at infant age of 6 weeks, 3 months and 5.5 months. The method involves the mother drinking an accurately measured 30-g dose of deuterium oxide (99.8% atom % purity, Sercon Ltd. UK, lot no. EB2039). Deuterium is a stable isotope of hydrogen, ie it is not radioactive and is considered safe and ethical for use in human studies and is also found in small quantities naturally in the body. No side effects have been noted at enrichment levels less than 0.2% and in this DTM method the deuterium enrichment level in the mother reaches a maximum of 0.1% in the body and less than half of that amount in the infant [19]. The dose of deuterium oxide given to the mother mixes rapidly with her body water and therefore also appears in her saliva and breast milk. If the infant is breastfed the deuterium oxide will pass through the breast milk to the infant and will then mix with the infant’s body water. The infant will thus have an enriched level of deuterium in its body water, which is sampled in its saliva. Saliva samples are taken from the mother and infant over a period of 14 days and the deuterium enrichment is measured in each sample using a Fourier Transform Infrared Spectrometer (FTIR) compared to their pre-dose saliva sample. Each saliva sample is measured twice and precision is high using this method, Coefficient of Variation (CV) values < 1% are achieved and enables differences in deuterium enrichment over time to be determined. The Solver function of Excel® is then used to fit the deuterium enrichment of the saliva samples from the mother and the infant over the 14-day period to model curves. This function minimizes the sum of the squares of the differences of the deuterium enrichments obtained from the FTIR and model values. This then yields values for breast milk intake (labeled water) and non-milk oral intake (NMOI, ie water from sources other than breast milk, which is unlabeled). An infant was determined to be exclusively breastfed according to the DTM method if the value of NMOI is ≤25 g/day [11]. A recent validation study [20] has proposed a higher cut off value of NMOI ≤82.6 g/day and therefore the results from the study are analyzed using both cut off values. The DTM method has been validated against the test weighing technique for measuring milk intake volumes and good correlations were observed [21]. It is a highly sensitive technique as deuterium enrichments in the infant’s saliva can only originate from the mother’s milk.

### Statistical analysis

A Kappa analysis was performed to validate reported infant feeding practice against the infant feeding practice as determined by the DTM method using STATA Version 13, © Statacorp, Texas, USA. Kappa analysis compares the observed level of agreement with the level of agreement expected by chance alone, with a scale of 0, representing an agreement that can be expected by chance alone, to 1.0 which would represent a perfect agreement (or − 1.0 a perfect disagreement) [22]. Further sub-classifications are: 0.01–0.20 slight agreement; 0.21–0.40 fair agreement; 0.41–0.60 moderate agreement; 0.61–0.80 substantial agreement and 0.81–1.00 almost perfect agreement.

## Results

All mothers were not available for all study time points, therefore at each time point the number of participants was less than the total of 100 mother-baby pairs that were recruited in total. At the six week time point, for example, only 47 mother-baby pairs were recruited due to a delay in ethics approval, which resulted in some participants only commencing the study at the three month time point.

The results were checked for missing entries and valid ranges. Any high or low values outside valid ranges were identified using scatter plots and histograms and as a result 1, 3, and 2 measurements were excluded at the 6 week, 3 month and 5.5 month time points, respectively. Measurements were also checked for a good fit to the model and this resulted in four and one values being excluded at the 3 and 5.5 months time points, respectively because of a poor fit to the model. These were mostly due to errors in sample collection or incomplete sample collection. The total number of measurements available after data cleaning was 46, 74 and 72 mother-infant pairs at 6 weeks, 3 months, and 5.5 months, respectively. There were two mother’s reports missing at three months, both were non-exclusively breastfeeding mothers according to the DTM method results. Three maternal reports were missing at 5.5 months, one was exclusively and two non-exclusively breastfeeding according to the DTM method.

**Fig. 1:**
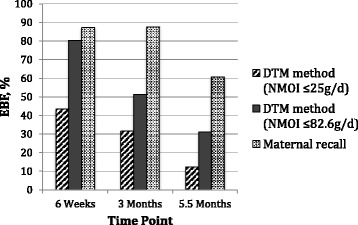
Comparison of exclusive breastfeeding rates from our study (DTM method and maternal recall). DTM: dose to mother, NMOI: Non-milk oral intake

Exclusivity of breastfeeding using the DTM method was determined using the two cut off values for NMOI and was compared to maternal recall results in Fig. 1.

Kappa analysis of the mother’s reported infant feeding compared to the infant feeding as determined by the DTM method yielded the results as shown in Table 1.

**Table 1:** Kappa analysis of exclusive breastfeeding from mother’s report vs DTM method using two different cut offs

Time point	Cut off for EBF, NMOI g/day	Observed agreement, %	Expected agreement, %	*p*-value	Kappa value
6 weeks	≤ 25	52.17	45.18	0.078	0.13
	≤ 82.6	76.09	72.50	0.181	0.13
3 months	≤ 25	36.49	36.79	0.525	0.00
	≤ 82.6	51.35	52.05	0.573	−0.01
5.5 months	≤ 25	49.30	40.73	0.009	0.14
	≤ 82.6	57.75	45.45	0.011	0.23

*DTM* dose to mother, *EBF* exclusive breastfeeding, *NMOI* non-milk oral intake

The results showed that there was only a “slight” or “fair” agreement (using the higher cut off value) between the two methods at 5.5 months and this was statistically different to the agreement expected by chance alone. Kappa values which were not statistically significant (ie could be caused by chance alone) showed only “slight” agreement at six weeks and a “less than chance” agreement between the two methods at the three month time point using both cut off values (< 0.01).

## Discussion

The rates of exclusive breastfeeding as determined by maternal recall found in our study are similar to the rates reported in two other South African studies where the mothers were receiving fairly intensive counseling and support for exclusive breastfeeding. The one South African study showed exclusive breastfeeding rates, based on mother’s report, of 81.4% in HIV-infected mothers and 92.9% in HIV-uninfected mothers at 6–8 weeks [23], which are similar to the values reported by the mothers in our study (87.2% at six weeks). The mothers who reported exclusively breastfeeding their infants at 3–4 months was lower than in our study, at 61.6% and 72.6% respectively at 3–4 months, compared to our 87.7% at three months. The second South African study [24] which included only HIV-infected women reported similar exclusive breastfeeding rates from the mother’s report at six months of 57.4% compared to our 60.8%. Their value at three months was slightly higher than in our study, 92.1% at three months compared to our value of 87.7%.

More recently a 2014 survey in KZN prior to the implementation of the KIBS project showed a lower EBF rate of 44.6% at 14 weeks (personal communication, C. Horwood). All the percentages of exclusive breastfeeding from maternal recall were higher than the reported national averages and higher than the 2014 provincial average. Hence a marked improvement in exclusive breastfeeding was noticeable which could be attributed to the breastfeeding counseling that the mothers were receiving as part of the study, as well as the policy changes undertaken by the South African Government to promote exclusive breastfeeding for all mothers since the Tshwane Declaration [3]. Since the completion of this study the recently published 2016 South African Demographic and Health Survey (SADHS) has also shown the impact of the breastfeeding promotion efforts in South Africa with much higher rates of exclusive breastfeeding than the 2003 survey (44.0% < 2 months; 28.2% 2–3 months; 23.7% 4–5 months; and 4.9% 6–8 months; or 32.0% over the first six months) [25].

The Kappa analysis showed that there was very poor correlation between the mother’s report of exclusive breastfeeding and the objective determination of exclusive breastfeeding by the DTM method even when using a higher cut off value for non-milk oral intake. Kappa values for six weeks were in the range of “slight agreement” between the two methods regardless of the cut off value used for the DTM method. At three months the observed agreement was lower than the agreement expected by chance alone which resulted in Kappa values close to zero using both cut offs resulting in a classification of a “less than chance agreement”. At 5.5 months the Kappa values improved to the “slight agreement” or “fair agreement” categories depending on the cut off value used. Reasons for these varying Kappa values could be that at 6 weeks more infants were EBF according to the objective method (80.4% using the higher cut off value for NMOI) and mothers would therefore most likely report exclusive breastfeeding. At three months the objectively determined EBF rate had dropped to 51.3% using the higher cut off value for NMOI and the social desirability bias [26] could have resulted in many mothers over-reporting EBF and thus the resultant near zero Kappa values for both cut offs. The improved Kappa values at 5.5 months could have been due to mothers now feeling more comfortable to report that their infants were no longer EBF at this later time point. It is however worth mentioning that none of the Kappa values reached a classification of “moderate”, “substantial” or “almost perfect” [22].

These results are similar to those obtained in countries such as Cameroon, where the authors stated that 75% of the mothers in their report who claimed to be exclusively breastfeeding were in fact not doing so according to the DTM results [14]. Similarly to their study, this present study also educated the mothers with regards to the benefits of breastfeeding and exclusive breastfeeding, hence it is possible that mothers would report best practice even if they themselves were not carrying it out. This social desirability bias which has also been reported in dietary self-reports [26] could also be relevant in reporting breastfeeding behavior according to expectations and best practice. It then calls into question the validity of even the accuracy of mother’s reports used for health surveys and shows the benefit of methods such as the DTM method which gives a more accurate unbiased assessment of infant feeding practices.

In India a similar large discrepancy was found between reported exclusive breastfeeding and the results from the DTM method [15]. At one month the DTM method results showed that only 56% of mothers were exclusively breastfeeding, whereas 100% claimed to be exclusively breastfeeding. At three months, the DTM method showed that 23% were exclusively breastfeeding, whereas 90% of mothers reported that they were exclusively breastfeeding. At six months 14% were exclusively breastfeeding according to the isotope technique but 36% mothers claimed to be exclusively breastfeeding. Similarly, in Botswana 80% of mothers reported that they were exclusively breastfeeding their infants at six months, yet the DTM method showed that none of these mothers were in fact exclusively breastfeeding their infants [13]. In a study in Bangladesh, about 13% in the reported exclusive breastfeeding group were classified as non-exclusively breastfed according to the DTM method [12]. In our population, this value was higher regardless of the cut off value for NMOI used, 53%, 68% and 82% (≤ 25 g/day cut off) and 18%, 48% and 59% (≤ 82.6 g/day cut off) mothers reported that they were exclusively breastfeeding but the DTM method showed that they were not exclusively breastfeeding at 6 weeks, 3 months and 5.5 months, respectively.

A review on the reliability and validity of infant feeding practices as determined by maternal recall concluded that although initiation and duration of breastfeeding could be considered reliable and valid, the age of introduction of other solids or liquids were not [27]. This study referred to retrospectively gathered data, which is commonplace in nationwide surveys and thus calls into question the validity of the surveys that are then used to inform policy making. The advantage of the DTM method for determining exclusive breastfeeding is that the results are objective and can lead to more accurate estimates of true exclusive breastfeeding rates. It is also non-invasive, and it does not interfere with the mother’s normal feeding pattern. However, there are drawbacks to the current method. These include the cost of deuterium and the FTIR; and the fact that seven samples must be taken from the mother and infant over a period of 14 days. The latter is labor intensive and costly in terms of staff time and logistics if home visits are undertaken, and therefore be unsuitable in its current format where large sample sizes are required. However, a promising new study has been undertaken where it is possible to take only two post-dose samples, compared to the current seven post-dose samples, in order to determine if an infant is exclusively breastfed [28] and this would make the DTM method more feasible logistically.

A limitation of the DTM method is that although it accurately assesses breast milk intake and non-milk oral intake over the 14 day period of observation, hence allowing EBF to be objectively determined, it may not be representative of breastfeeding practices outside this 14 day period. Breastfeeding patterns are not necessarily constant over time and a mother can change from a period of mixed feeding to exclusive breastfeeding when given infant feeding support. However, the same is also true of the 24 h maternal recall, which may not be valid for another period of time.

The cut off value for non-milk oral intake, which had been originally set at ≤ 25 g/day for the DTM method, has now been further investigated and the new higher cut off value may represent the actual value of exclusively breastfed infants more accurately [20]. However, both values of cut off show that there is still an overestimation of exclusive breastfeeding using maternal recall compared to the DTM method.

## Conclusions

Maternal recall of EBF is shown to be limited in terms of accuracy when compared to the gold standard DTM method and thus the latter would be recommended for determining the impact of an intervention with smaller representative samples. The current logistics of seven sampling visits make it unsuitable for use in large and long-term epidemiological surveys and routine clinic assessments, however new developments in the methodology may make this objective method more feasible and result in a more accurate representation of exclusive breastfeeding rates.

## Abbreviations

DTM: Dose to mother
EBF: Exclusively breastfed
FTIR: Fourier Transform Infrared
HIV: Human Immunodeficiency Virus
IAEA: International Atomic Energy Agency
KIBS: KwaZulu-Natal Initiative for Breastfeeding Support
KZN: KwaZulu-Natal
NMOI: Non-milk oral intake
SADHS: South African Demographic and Health Survey
WHO: World Health Organization

## References

[1] Rollins NC, Bhandari N, Hajeebhoy N, Horton S, Lutter CK, Martines JC (2016). Why invest, and what it will take to improve breastfeeding practices?. Lancet.

[2] South African Demographic and Health Survey 2003*:* Full report, National Department of health, Pretoria, South Africa 2004.

[3] The Tshwane Declaration of Support for Breastfeeding in South Africa. Department of health, Tshwane, South Africa, 2011.

[4] KZN Department of Health media release: “KZN Department of Health in R32 million drive to support and encourage breastfeeding in KZN, media release”, http://www.kznhealth.gov.za/mediarelease/2014/KIBS_0408201.htm (accessed 01 Nov 2017).

[5] National Department of Health (2013). The National Antenatal Sentinel HIV Prevalence Survey South Africa.

[6] World Health Organization 2010: Guidelines on HIV and infant feeding, Geneva, Switzerland.

[7] National Department of Health (2010). National Department of health South Africa and south African national AIDS council: clinical guidelines: PMTCT (prevention of mother-to-child transmission).

[8] National Department of Health (2017). Technical Update: 2013 Infant and Young Child Feeding (IYCF) Policy amendment South Africa.

[9] World Health Organization (2014). Global nutrition targets 2025 breastfeeding policy brief.

[10] Coward WA, Cole TJ, Sawyer MB, Prentice AM (1982). Breast-milk intake measurement in mixed-fed infants by administration of deuterium oxide to their mothers. Human Nutr Clin Nutr.

[11] Stable Isotope Technique to Assess Intake of Human Milk in Breastfed Infants, Human Health Series No. 7, Published Vienna 2010. International Atomic Energy Agency (IAEA) website, http://www-pub.iaea.org/books/iaeabooks/8168/Stable-Isotope-Technique-to-Assess-Intake-of-Human-Milk-in-Breastfed-Infants (accessed 07 November 2017).

[12] Moore SE, Prentice AM, Coward WA, Wright A, Frongillo EA, Fulford AJC (2007). Use of stable-isotope techniques to validate infant feeding practices reported by Bangladeshi women receiving breastfeeding counseling. Am J Clin Nutr.

[13] Motswagole BS, Matenge STP, Mongwaketse T, Bogopa J, Kobue-Lekalake R, Mosetlha K (2015). Application of the deuterium-oxide dose-to-mother technique to determine the exclusivity of breasfeeding in women in Kanye, Botswana. S Afr J Clin Nutr.

[14] Medoua GN, Sajo Nana EC, Ndzana ACA, Makamto CS, Etame LS, Rikong HA (2012). Breastfeeding practices of Cameroonian mothers determined by dietary recall since birth and the dose-to-the-mother deuterium-oxide turnover technique. Matern Child Nutr.

[15] Samuel TM, Thomas T, Bhat S, Kurpad AV (2012). Are infants born in baby-friendly hospitals being exclusively breastfed until 6 months of age?. Eur J Clin Nutr.

[16] Mulol H, Coutsoudis C (2016). Breastmilk output in a disadvantaged community with high HIV prevalence as determined by the deuterium oxide dose-to-mother technique. Breastfeed Med.

[17] WHO Indicators for assessing breastfeeding practices. Division of child health and development, Geneva, Switzerland, 1991.

[18] World Health Organization. Indicators for assessing infant and young child feeding practices*.* Part 1 Definitions. Geneva, Switzerland 2008.

[19] Jones PJ, Leatherdale ST (1991). Stable isotopes in clinical research: safety reaffirmed. Clin Sci (Lond).

[20] Diana A, Liu Z, Luftimas D, Rahmannia S, Preston T, Slater C (2017). Development of a non-milk water intake cutoff to identify exclusive breastfed infants using the deuterium oxide dose-to-mother technique. Ann Nutr Met..

[21] Butte NF, Wong WW, Patterson BW, Garza C, Klein PD (1988). Human-milk intake measured by administration of deuterium oxide to the mother: a comparison with the test-weighing technique. Am J Clin Nutr.

[22] Viera AJ, Garrett JM (2005). Understanding interobserver agreement: the kappa statistic. Fam Med.

[23] Rollins NC, Ndirangu J, Bland RM, Coutsoudis A, Coovadia HM, Newell ML (2013). Exclusive breastfeeding, diarrhoeal morbidity and all-cause mortality in infants of HIV-infected and HIV uninfected mothers: an intervention cohort study in KwaZulu Natal, South Africa. PLoS One.

[24] Kindra G, Coutsoudis A, Esposito F, Esterhuizen T (2012). Breastfeeding in HIV exposed infants significantly improves child health: a prospective study. Matern Child Health J.

[25] South African Demographic and Health Survey 2016: key indicator report, Statistics South Africa, National Department of health, Pretoria, South Africa 2017.

[26] Herbert JR, Hurley TG, Petersen KE, Resnicow K, Thompson FE, Yaroch AL, et al. Social desirability trait influences on self-reported dietary measures among diverse participants in a multicenter multiple risk factor trial. J Nutr. 2008:226S–34S. 10.1093/jn/138.1.226S 18156429

[27] Li R, Scanlon KS, Serdula MK (2005). The validity and reliability of maternal recall of breastfeeding practice. Nutr Rev.

[28] Houghton L, Lui Z, Preston T, Slater C, Diana A, Gibson R (2017). Development and evaluation of a reduced protocol for the deuterium oxide dose-to-mother technique to assess exclusive breastfeeding practices. Ann Nutr Met.

